# The essential roles of OsFtsH2 in developing the chloroplast of rice

**DOI:** 10.1186/s12870-021-03222-z

**Published:** 2021-10-01

**Authors:** Qingfei Wu, Tiantian Han, Li Yang, Qiang Wang, Yingxian Zhao, Dean Jiang, Xiao Ruan

**Affiliations:** 1 School of Biological and Chemical Engineering, NingboTech University, Ningbo, 315100 China; 2 Ningbo Research Institute, Zhejiang University, Ningbo, 315100 China; 3 State Key Laboratory of Plant Genomics, National Centre for Plant Gene Research (Beijing), Institute of Genetics and Developmental Biology, Chinese Academy of Sciences, Beijing, 100101 China; 4 State Key Laboratory of Plant Physiology and Biochemistry, College of Life Sciences, Zhejiang University, Hangzhou, 310058 China

**Keywords:** Rice, OsFtsH2, Chloroplast development, RNA sequencing

## Abstract

**Background:**

Filamentation temperature-sensitive H (FtsH) is an ATP-dependent zinc metalloprotease with ATPase activity, proteolysis activity and molecular chaperone-like activity. For now, a total of nine FtsH proteins have been encoded in rice, but their functions have not revealed in detail. In order to investigate the molecular mechanism of *OsFtsH2* here, several *osftsh2* knockout mutants were successfully generated by the CRISPR/Cas9 gene editing technology.

**Results:**

All the mutants exhibited a phenotype of striking albino leaf and could not survive through the stage of three leaves. OsFtsH2 was located in the chloroplast and preferentially expressed in green tissues. In addition, *osftsh2* mutants could not form normal chloroplasts and had lost photosynthetic autotrophic capacity. RNA sequencing analysis indicated that many biological processes such as photosynthesis-related pathways and plant hormone signal transduction were significantly affected in *osftsh2* mutants.

**Conclusions:**

Overall, the results suggested OsFtsH2 to be essential for chloroplast development in rice.

**Supplementary Information:**

The online version contains supplementary material available at 10.1186/s12870-021-03222-z.

## Background

As the sites of photosynthesis to take place in plants, chloroplasts are unique organelles to capture and transform light energy into chemical energy, and thus provide energy for plant growth and development [1]. Also chloroplasts are responsible for the biosynthesis of various metabolites including tetrapyrroles, amino acids, lipids, terpenoids, hormones, etc. [2]. As a semi-autonomous organelle, chloroplast has its own DNA genome and protein synthesis machinery, but only a part of the proteins can be synthesized in chloroplast, and most proteins are synthesized on ribosomes of cytoplasm [3]. The development of chloroplast is a complex process modulated by both the plastid and nuclear genes, which can be divided into three steps [4]. The first step is to activate replication and DNA synthesis of plastid. The second step is the “build up” of chloroplast, characterized by the establishment of chloroplast genetic system, in which plastid genes to encode the gene expression machineries are preferentially transcribed by NEP (nuclear encoded plastid RNA polymerase), and the transcription / translation activity in chloroplast is strikingly increased [5]. In the third step, the plastid and nuclear genes encode photosynthetic apparatus are massively expressed, the plastid genes are principally transcribed by PEP (plastid-encoded RNA polymerase) [6], and expression of these genes results in chloroplast biosynthesis and assembly. Up to now, however, the molecular mechanism to regulate the development of chloroplasts in higher plants remain largely unknown [7].

Filamentation temperature-sensitive H (FtsH) is an ATP-dependent zinc metalloprotease, which exists widely in eukaryotes (mitochondria and chloroplasts) and prokaryotes [8, 9]. It is a member of the AAA (ATPase associated with diverse cellular activities) protein family, and possesses ATPase activity, proteolysis activity and molecular chaperone-like activity [10]. In general, FtsH protein contains tow transmembrane α -helices at the N terminus, which anchor the protein to the membrance of thylakoid or mitochondria. The C terminus of FtsH protein is located in the cytoplasm, consisting of an AAA-type domain and a M41-like endoprotease domain. FtsH was firstly found in *Escherichia coli* as an essential gene, which mediated various processes including the exporting of proteins from cell, membrane modeling, protein quality control to resist colicin and mRNA decay [11–13]. While FtsH is encoded by only a single gene in most prokaryotes genomes, multiple isoforms are identified in algae, cyanobacteria and plants [14]. It has been proposed that the multiplication of FtsH genes is related to the evolution of oxygenic photosynthesis and this trend is maintained in higher plants [15]. For example, four homologous genes of FtsH have been found in the cyanobacterium, *Synechocystis sp. PCC 6803* [16], and among them, *slr0228* was reported to play vital roles in the degradation of the photodamaged D1 proteins and removing the unassembled PSII subunits from the thylakoid membrane [17, 18]. In the soybean genome, a total of 11 FtsH genes have been identified, in which *GmFtsH9* could be involved in regulating PSII function [19].

A total of 12 FtsH proteins have been encoded in *Arabidopsis thaliana.* Among them, three members (AtFtsH3, AtFtsH4, and AtFtsH10) are targeted to mitochondria and eight members (AtFtsH1, AtFtsH2, AtFtsH5 to AtFtsH9, and AtFtsH12) to chloroplasts, while AtFtsH11 appears to be dual-targeted to both mitochondria and chloroplasts [20, 21]. Under normal conditions of growth, *AtFtsH2* (also termed *VAR2*) is the most abundantly expressed gene, followed by *AtFtsH5* (also termed *VAR1*), *AtFtsH8* and *AtFtsH1,* and the others are expressed at very low levels [22]. In addition, chloroplastic FtsHs predominantly form a hetero-hexameric complex comprising at least two types of isomers, type A (*AtFtsH1/5*) and type B (*AtFtsH2/8*) which are functionally distinguishable from each other [23, 24]. Both AtFtsH1/AtFtsH5 and AtFtsH2/AtFtsH8 proteases have been confirmed to participate in degradation of the photodamaged PSII D1 protein [25–27] and unassembled thylakoid membrane proteins [28, 29]. Disruption of *AtFtsH2* results in a severe leaf variegation phenotype (*var2*) and disruption of *AtFtsH5* results in a weak leaf variegation phenotype (*var1*), but *AtFtsH1* and *AtFtsH8* mutants have no visible phenotypes [21, 30–32]. Meanwhile, the double mutants of *Atftsh1Atftsh5* and *Atftsh2Atftsh8* show an albino-like phenotype, suggesting that each subunit is required for chloroplast biogenesis [28, 33]. The mitochondrial *AtFtsh3*, *AtFtsh4* and *AtFtsh10* play crucial roles in the assembly/stabilization of the mitochondrial complexes, and the activities of mitochondrial complexes I and V are significantly reduced in these mutants [34]. Moreover, those mutants lacking of *AtFtsH4* show severe abnormal development of late rosette leaves, accompanied by ultrastructural impairment in chloroplasts and mitochondria [35]; *AtFtsH6* will contribute to the degradation of the light-harvesting complex of PSII under conditions of high light and senescence [36], and can also restrict the thermomemory by regulating HSP21 protein abundance [37]. Furthermore, the mutation of *AtFtsH11* causes a significant decrease in photosynthetic capability when environmental temperature raise above optimal, indicating its essential role in maintaining the thermostability in *Arabidopsis* plants [38–40], and also a recent study reported that the modulation of AtFtsH12 abundance causes an altered composition of the plastid import machinery, which will affect development of functional photosynthetic chloroplast [41]. All of the above research results suggest that the FtsH gene family participates in multiple processes in *Arabidopsis,* but the detailed molecular mechanisms need to be further studied.

So far nine FtsH proteins have been encoded in the genome of rice (*Oryza sativa*) [28, 42], including three members of OsftsH3, OsftsH4 and OsftsH5 to be predictably targeted to mitochondria, and the others to chloroplasts [42]. However, there are few reports on the functions and molecular mechanism of FtsH genes in rice. In this study, the knockout mutants of *OsFtsH2* with an albino seedling phenotype were generated by CRISPR/Cas9 gene editing technology, and the molecular mechanism of OsFtsH2 was explored. By combining a phenotypic and RNA sequencing analysis, it was found that OsFtsH2 could play a vital role in the chloroplast development.

## Results

### Identification and sequence analysis of OsFtsH2

**Fig. 1:**
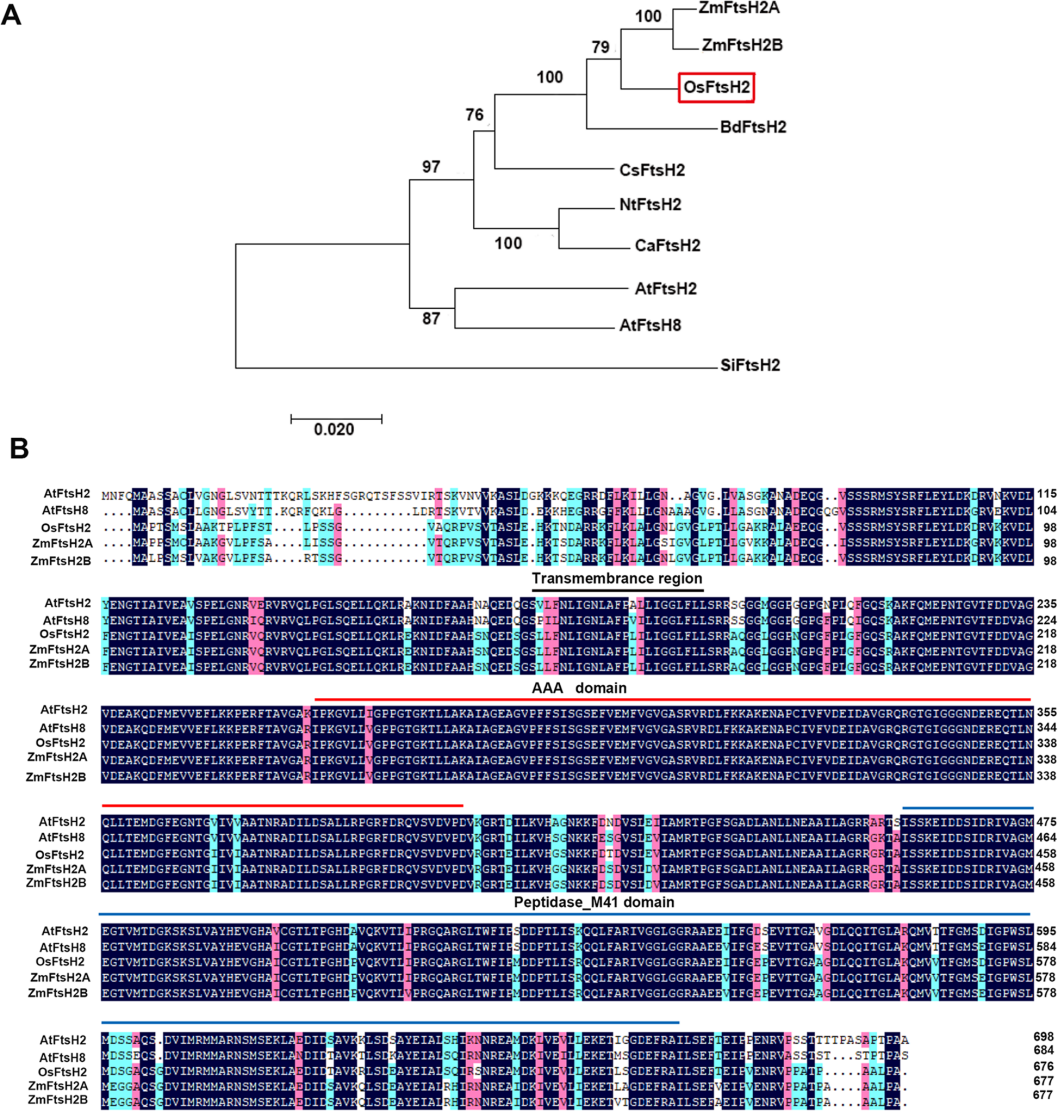
Phylogenetic and sequence analyses of OsFtsH2. **A** Phylogenetic analysis of OsFtsH2 and its closely related FtsH proteins from other species. The accession numbers of selected FtsHs are as follows: from *Arabidopsis thaliana*, AtFtsH2 (NP_850156.1) and AtFtsH8 (NP_001321589.1); from *Oryza sativa*, OsFtsH2 (XP_015643053.1); from *Zea mays*, ZmFtsH2A (NP_001120720.1) and ZmFtsH2B (NP_001120721.1); from *Brachypodium distachyon*, Bdftsh2 (XP_010240568.1); from *Nicotiana tabacum*, NtFtsH2 (XP_016436682.1); from *Capsicum annuum*, CaFtsH2 (XP_016580880.1); from *Cucumis sativus*, CsFtsH2 (XP_004136837.1); from *Setaria italica*, SiFtsH2 (XP_004966311.1). **B** Multiple sequence alignment of amino acid sequences of OsFtsH2 proteins with other FtsH2 proteins from various plants

Among the nine members of the FtsH family identified in rice genome [28], sequence analysis showed that *OsFtsH2* (LOC_Os01g43150) contained a complete ORF of 2031 bp and encoded a protein of 676 amino acids. Here a phylogenetic tree including ten FtsH2 proteins in rice and other plants was constructed (Fig. 1), and phylogenetic analysis showed that OsFtsH2 was clustered more closely with ZmFtsH2A and ZmFtsH2B than other FtsH2 proteins (Fig. 1A). Besides, OsFtsH2 protein harbors the conserved domains such as transmembrane region, AAA domain and peptidase M41 region, similar to its counterparts in plant species (Fig. 1B).

### Expression pattern and subcellular localization of OsFtsH2

**Fig. 2:**
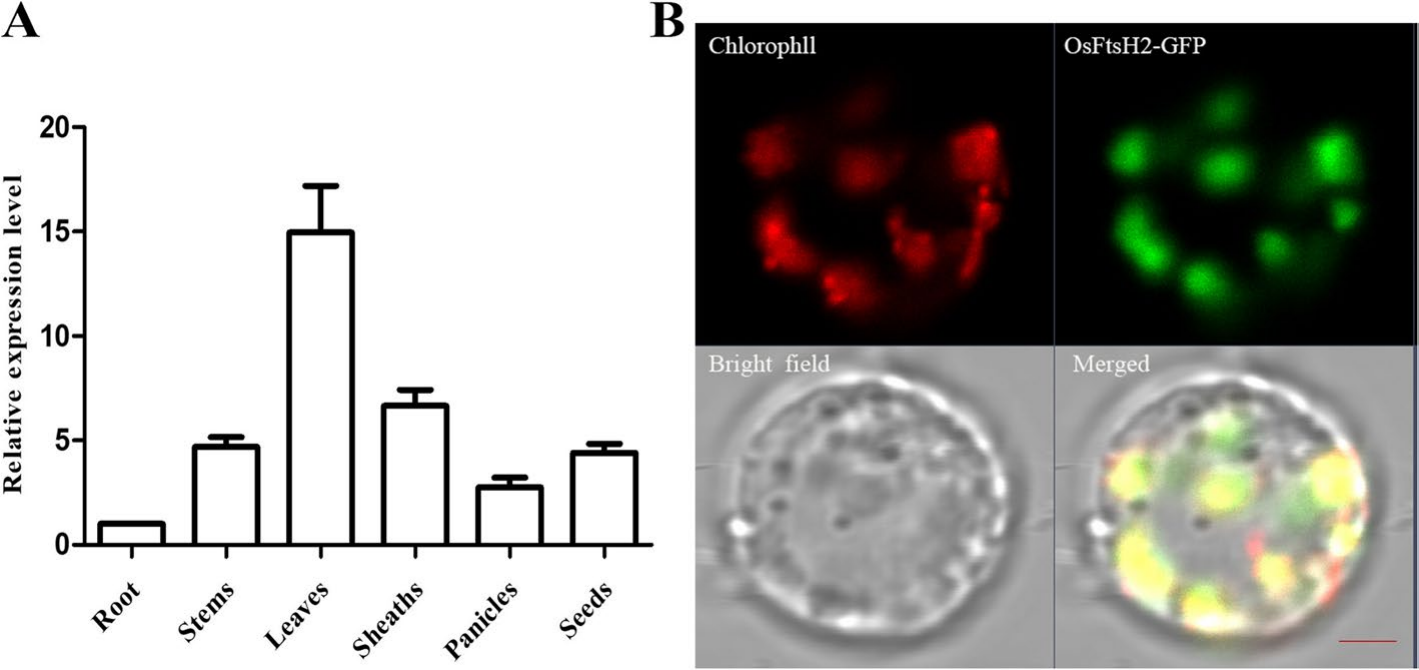
Expression pattern and subcellular localization of OsFtsH2. **A** Expression analysis of the *OsFtsH2* gene in different tissues. **B** Subcellular localization of the OsFtsH2 proteins. GFP signals of OsFtsH2-GFP fusion protein was located in the chloroplasts by transient expression analyses in rice protoplasts. Green fluorescence shows GFP, red fluorescence shows chloroplast auto-fluorescence and yellow fluorescence shows the merged fluorescence. Scale bar, 5 μm

Expression of genes in various tissues may display functional diversity. To determine expression pattern of *OsFtsH2*, RNA was extracted from roots, leaves, stems and panicle of wild type plants, and then implemented its reverse transcription to cDNA. The expression levels of *OsFtsH2* in these tissues were assessed by qRT-PCR. As shown in Fig. 2A, leaves revealed the highest level of *OsFtsH2* expression, followed in turn by sheaths, stems, seeds, panicles and roots. Therefore, *OsFtsH2* mainly functions in green tissues, just as predicted to be a chloroplast-targeted protein [42]. To verify the precise subcellular localization of OsFtsH2, the fusion vector of 35S: OsFtsH2-GFP was constructed and transiently expressed in rice protoplasts (Fig. 2B). As seen, the green fluorescent signals of OsFtsH2-GFP fusion proteins overlapped with chloroplast auto-fluorescence in transformed rice protoplasts, indicating that OsFtsH2 was localized in chloroplasts.

### The albino seedling phenotype caused by knockout of *OsFtsH2*

**Fig. 3:**
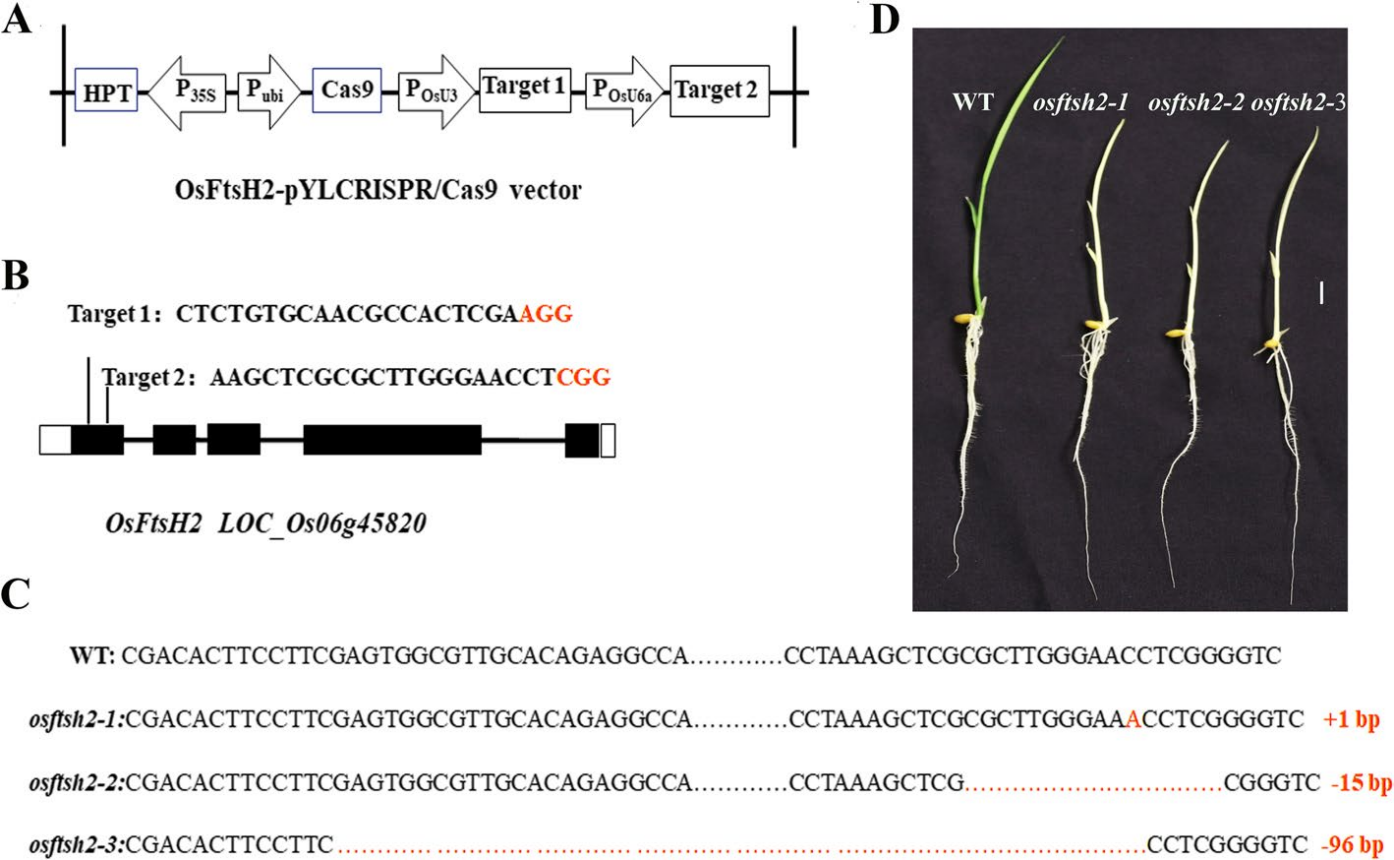
Production of *OsFtsH2* knockout mutants via the CRISPR/Cas9 system. **A** Diagram of CRISPR/Cas9 system for editing *OsFtsH2*. **B** Schematic diagram of targets sites in *OsFtsH2*. Black boxes show exons, black lines show introns and white boxes show untranslated regions (UTR). **C** Mutation sites of *OsFtsH2* knockout lines. The *osftsh2–1* mutant has a 1-bp insertion; the. *osftsh2–2* mutant has a 15-bp deletion; and the *osftsh2–3* mutant has a 96-bp deletion. **D** Phenotypes of *osftsh2* mutants, 7-day-old seedlings were photographed. Scale bar, 1 cm

**Fig. 4:**
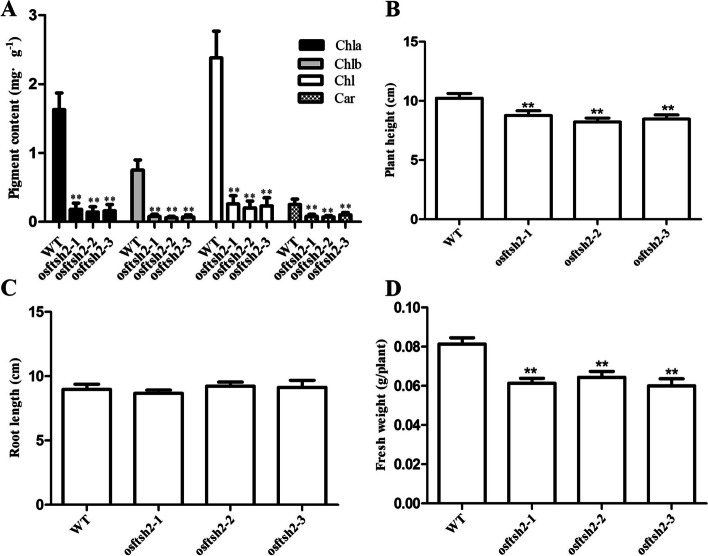
Characteristics of *osftsh*2 mutants at 7-day-old seedling stage. **A** Pigment content of WT and *osftsh*2 mutants. Chlorophyll a (Chla), chlorophyll b (Chlb), total chlorophyll (Chl) and carotenoid (Car). **B** Plant height of wild type and *osftsh*2 mutants. **C** Root length of WT and *osftsh*2 mutants. **D** Fresh weight of WT and *osftsh*2 mutants. The data are mean ± SD (*n* = 3) and ** indicates statistical significance at *p* < 0.01

To further understand the function of *OsFtsH2*, a CRISPR/Cas9 vector with carrying two target sites in the first exon of *OsFtsH2* was constructed (Fig. 3A, B). Then, the plasmid was integrated into the calli of wild-type rice (Donjin) by Agrobacterium-mediated transformation. For the T_2_ generation, sequencing analysis showed three transgene-free homozygous knockout lines of *osftsh2–1*, *osftsh2–2* and *osftsh2–3* (Fig. 3C), and all three of mutants exhibited the albino leaf phenotypes (Fig. 3D). Consistent with their phenotypes, photosynthetic pigment contents of the three *osftsh2* mutants were dramatically reduced as compared with that of the wild type (Fig. 4A). In addition, the plant height and seedling fresh weight of these *osftsh2* mutants were much lower than that of wild type, while there was no visible difference in their root lengths (Fig. 4B, C, D). It was also observed that the *osftsh2* mutants could not survive through the stage of three leaves.

### Photosynthetic characteristics of *osftsh2* mutants

**Fig. 5:**
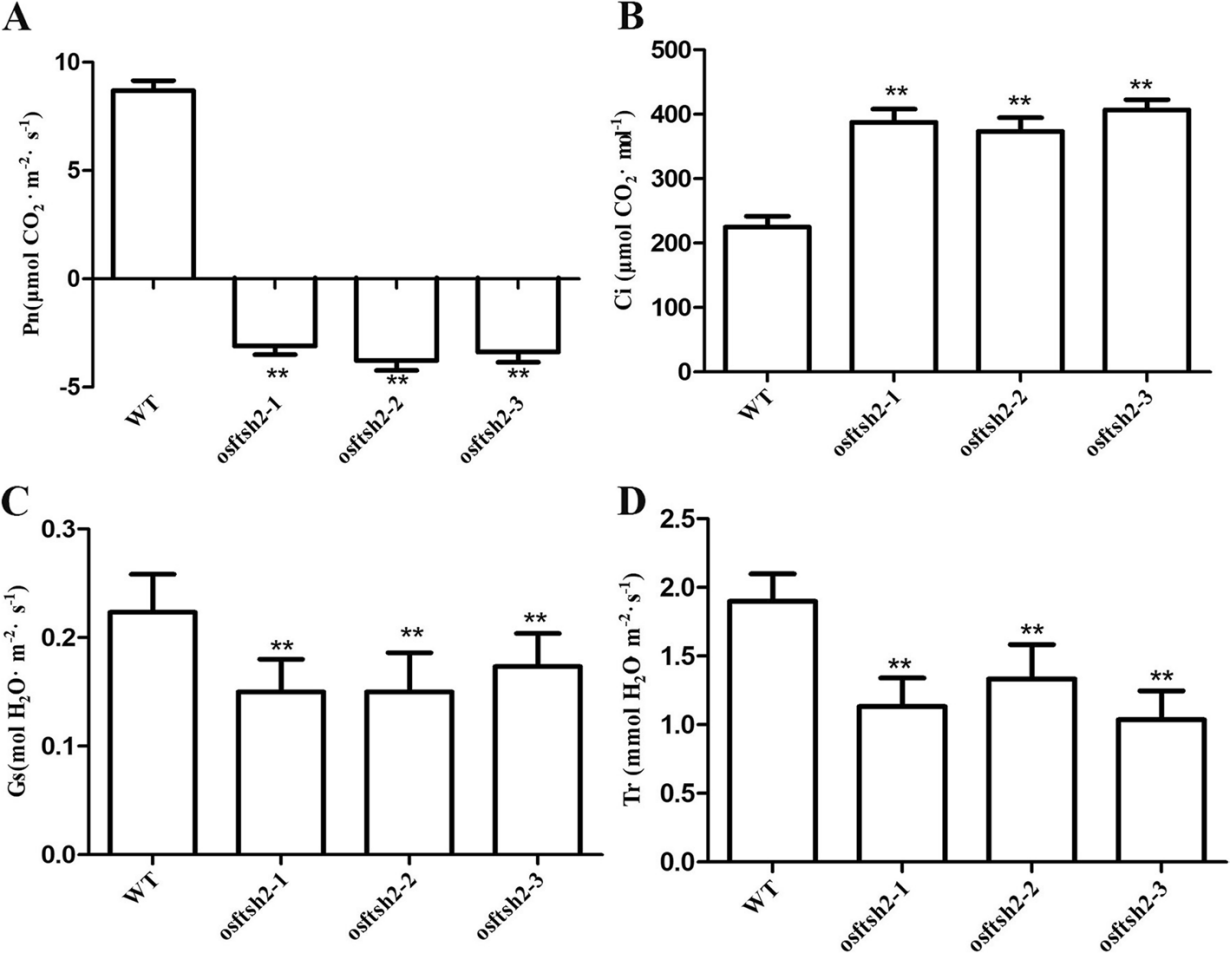
Measurement of leaf photosynthetic parameters in WT and *osftsh2* mutants at the seedling stage. **A** Pn, the net photosynthetic rate; **B** Ci, the intercellular CO_2_ concentration; **C** Gs, the stomatal conductance; **D** Tr, the transpiration rate. The data are mean ± SD (n = 3) and ** indicates statistical significance at *p* < 0.01

With a non-invasive feature of photosynthesis, chlorophyll fluorescence has been extensively used to monitor the changes in physiological state of photosynthetic apparatus [43]. The fluorescence analysis showed that the values of Fv/Fm in wild type rice and the *osftsh2* mutant were 0.80 and 0.36, respectively, and the actual photochemical efficiency (ФPSII and ФPSI) of the *osftsh2* mutant was also reduced significantly as compared with that of wild type (Fig. S1). In order to further determine the photosynthesis changes in *osftsh2* mutants, the photosynthetic parameters of the wild type and its mutants were measured. Compared with wild type plants as shown in Fig. 5, the net photosynthetic rate (Pn) of wild type was about 9.14 μmol CO_2_ m^− 2^ s^− 1^ but those of the *osftsh2* mutants dropped down to negative domain (Fig. 5A); the intercellular CO_2_ concentration (Ci) of each mutant was significantly higher than that of wild type (Fig. 5B); both stomatal conductance (Gs) and transpiration rate (Tr) of the *osftsh2* mutants were lower than that of the wild type (Fig. 5C and D). In brief, these results indicate that the light energy harvest and transfer were seriously blocked in *osftsh2* mutants, which would cause the loss of their autotrophic ability of photosynthesis.

### Impairment of chloroplast development in *osftsh2* mutants

**Fig. 6:**
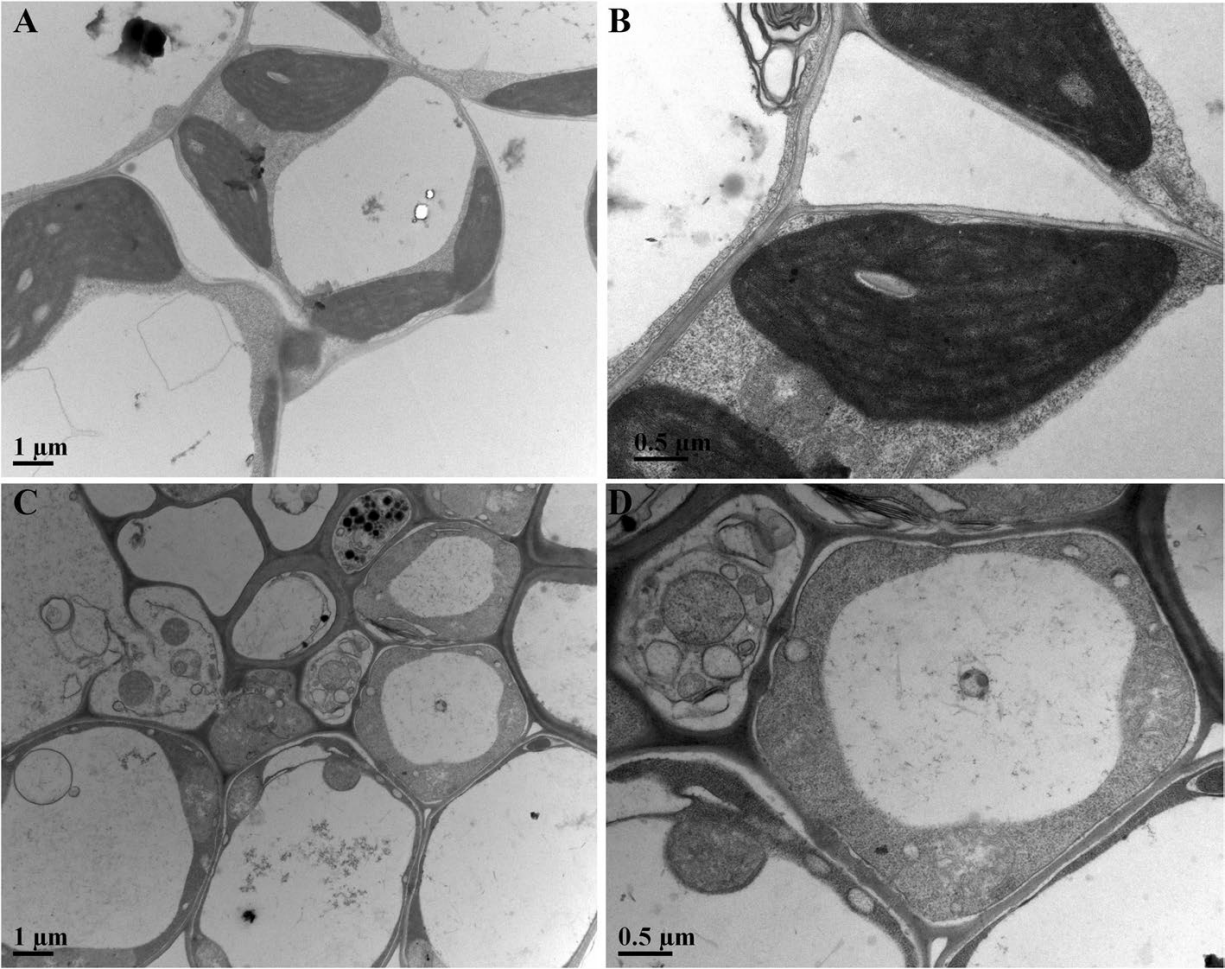
Transmission electron microscopic images of chloroplasts in WT (**A**, **B**) and osftsh2 mutants (**C**, **D**)

Chloroplast was developed by successive biosynthesis and assembly of chlorophyll into photosynthetic apparatus [44]. The albino leaf phenotypes (Fig. 3D) displayed the unhealthy development of chloroplast in *osftsh2* mutants. For further verification, the ultrastructure of chloroplasts in both wild type and *osftsh2–1* mutant leaves was analyzed by transmission electron microscopy (Hitachi H-7650). As illustrated in Fig. 6, the chloroplasts in wild type showed normal shape and contained a large number of well-structured and dense grana stacks (Fig. 6A, B), and in contrast, the *osftsh2* mutant only revealed the mesophyll cells with vesicle-like structures instead of regular chloroplasts with visible grana lamellae stacks (Fig. 6C, D). These results demonstrated that the development of chloroplast in the *osftsh2* mutant was severely impaired.

### Accumulation of reactive oxygen species (ROS) in *osftsh2* mutants

**Fig. 7:**
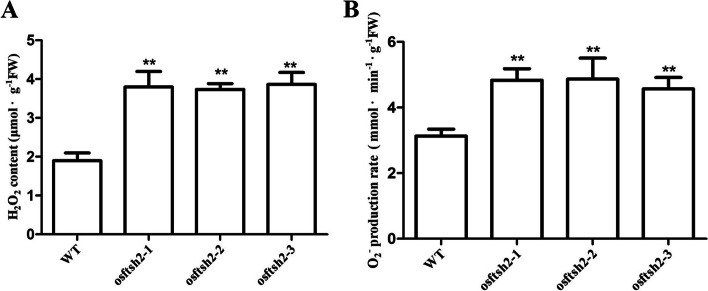
Production of reactive oxygen species in WT and *osftsh*2 mutants. **A** Contents of hydrogen peroxide. **B** Production rate of superoxide. The data are mean ± SD (n = 3) and ** indicates statistical significance at p < 0.01

FtsH proteins play important roles in photo-oxidative stress, which can participate in degradation of photodamaged D1 protein [24]. ROS levels in chloroplasts were always increased under photo-oxidative stress, and function as signaling molecules to regulate chloroplast-to-nucleus signal transduction [45, 46]. Therefore, we further determined the ROS levels in *osftsh2* mutants. As shown in Fig. 7, the contents of H_2_O_2_ and O_2_^–^ in *osftsh2* mutants were significantly increased as compared to that in wild type, indicating that the *osftsh2* mutants may suffer photo-oxidative damage due to the accumulation of excessive ROS.

### Analysis of differentially expressed genes (DEGs) in *osftsh2* mutants

In order to unravel the molecular mechanism of *OsFtsH2*, transcriptome analysis of wild type plants and *osftsh2* mutants were comparatively conducted with RNA-seq. In detail, total RNA was firstly extracted from the second leaves of wild type and *osftsh2–1* mutants at three leaves stage. Then, three biological replicates were conducted for each sample and a total of six libraries were constructed. High-throughput sequencing generated 46.31–56.69 million raw reads per library, and after a stringent quality filtering process, 45.89–56.14 million clean reads were obtained for each library (Table 1). About 98% of the Q20 percentage and 95% of the Q30 percentage were observed from RNA sequencing data, and the calculated GC content of each library showed that the average GC content was in the range of 53.16–54.97% (Table 1). Moreover, about 96% of the clean reads can be mapped to the rice reference genome, and more than 89% of the reads were uniquely mapped to the genome in each sample. Overall, the quality of sequencing results was qualified for further transcriptome analyses. In addition, the level of gene expression was characterized by calculating RPKM (reads per kb per million reads) values, and a total of 38,866 expressed genes with a RPKM > 1 value were detected from the RNA-seq data. By the end, a total of 6461 DEGs were identified in wild type or *osftsh2* mutants as examined with the criteria |log2(fold change) | > 1 and *p*-value < 0.05, and in *osftsh2* mutants there were 3226 significantly up-regulated genes and 3235 down-regulated genes, respectively (Table S1).

**Table 1:** Summary of RNA-sequencing results and quality data output

Sample	Raw reads	Raw bases	Clean reads	Clean bases	Q20(%)	Q30(%)	GC content (%)
WT_1	50207030	7581261530	49707036	7391192962	98.86	96.24	54.37
WT_2	56669228	8557053428	56146190	8368829529	98.86	96.24	54.97
WT_3	52257554	7890890654	51741726	7701808973	98.81	96.07	53.77
ftsh2_1	53626810	8097648310	53100052	7907867997	98.76	95.97	53.6
ftsh2_2	54130992	8173779792	53613774	7974079191	98.83	96.17	53.48
ftsh2_3	46312092	6993125892	45893594	6808826659	98.27	94.53	53.16

### Functional annotations and classifications of the DEGs

**Fig. 8:**
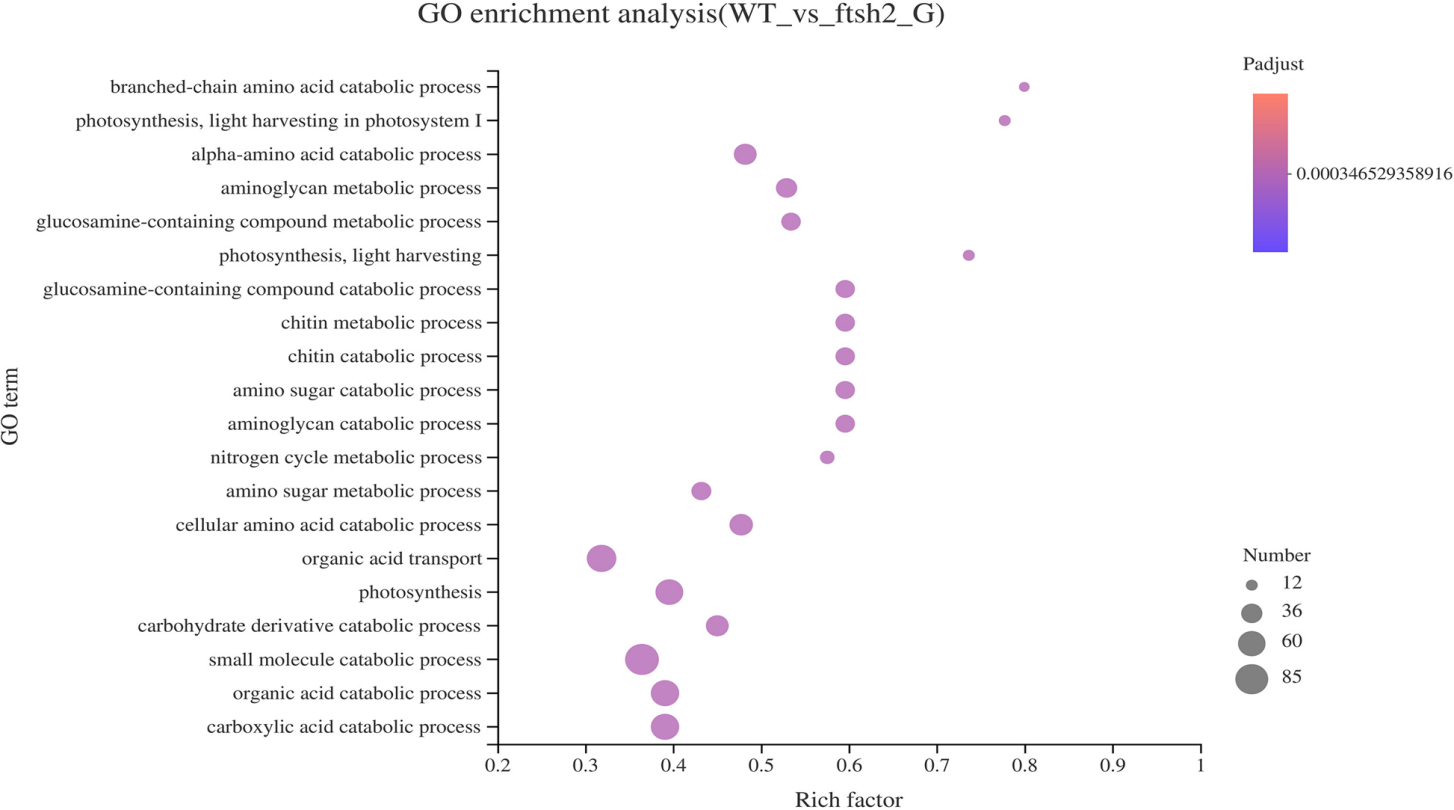
GO enrichment analysis of differentially expressed genes (DEGs) in *osfstsh2* mutants compared with WT

The DEGs were annotated and enriched in three sets of ontologies including molecular function (MF), cellular component (CC) and biological process (BP) based on GO database. The DEGs annotated in MF category included binding, catalytic activity, transporter activity, nucleic acid binding transcription factor activity and 9 other GO terms; the DEGs in CC consisted of 16 GO terms such as cell, cell part, organelle, membrane and macromolecular complex; also the DEGs in BP involved metabolic process, cellular process, single-organism process, biological regulation, response to stimulus and 16 other GO terms (Fig. S2). Furthermore, the top 20 most significantly enriched GO terms of these functional DEGs were shown in Fig. 8, notifying that no term of MF or CC categories emerged. In other words, only those terms in BP category such as branched-chain amino acid catabolic process, light harvesting in photosystem I, alpha-amino acid catabolic process and amino glycan metabolic process were enriched, suggesting that they were significantly influenced by *OsFtsH2*.

**Fig. 9:**
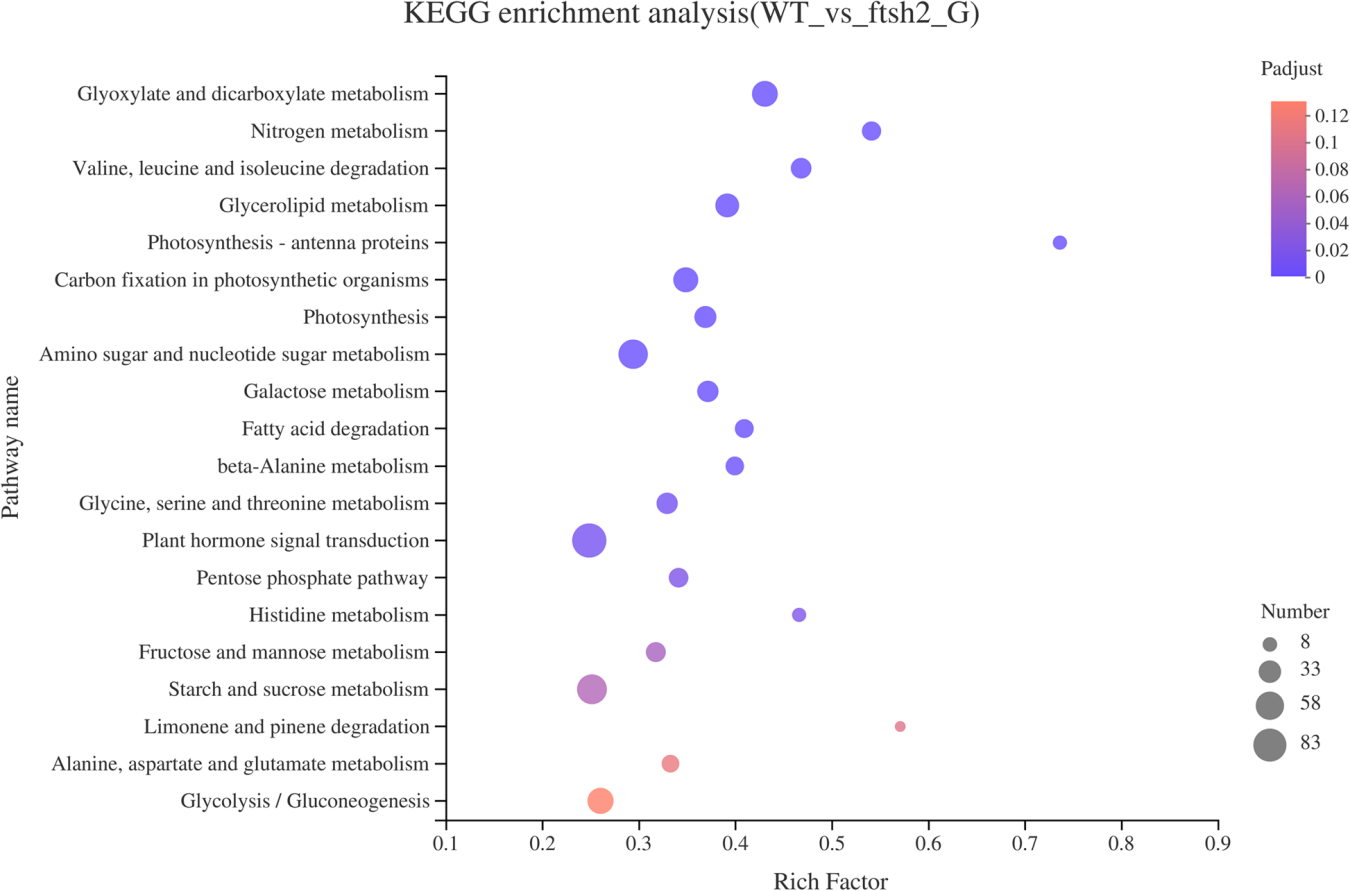
KEGG enrichment analysis of DEGs in *osfstsh2* mutants compared with WT

To determine the effect of *OsFtsH2* on the signal transduction pathways and metabolic mechanism in rice, KEGG (Kyoto Encyclopedia of Genes and Genomes) pathway analysis of DEGs in *osftsh2* mutants was conducted. In the first, the DEGs were classified into 138 pathways (Table S2), and then KEGG pathway enrichment analysis of DEGs was performed using Fisher’s exact test and the pathway with Padjust < 0.05 was considered to be significantly enriched. As a result, the top 20 most significantly enriched KEGG pathways were identified (Fig. 9), which include glyoxylate and dicarboxylate metabolism, nitrogen metabolism, plant hormone signal transduction, glycolysis / gluconeogenesis, etc. Overall, it was found that *OsFtsH2* was actively involved in regulating various pathways in rice.

### Suppression of photosynthetic genes in *osftsh2* mutants

As known, FtsH could participate in the progressive degradation of chloroplast proteins along with other proteases, and a comparative microarray analysis showed that numerous photosynthetic genes were repressed strongly in the *var2* white sectors [47]. According to the pathway enrichment analysis in this work, the photosynthesis-related pathways involving photosynthesis (34 genes), photosynthesis-antenna (14 genes) and carbon fixation in photosynthetic organisms (44 genes) were significantly enriched, and almost all genes of photosystem subunits and chlorophyll a-b binding protein were greatly down-regulated in *osftsh2* mutants (Table S2). Consequently, suppression of these photosynthetic genes could cause chloroplast defects in *osftsh2* mutants.

### Changes of plant hormone signal transduction in *osftsh2* mutants

Plant hormones play distinctive roles in controlling plant growth and development. It is worth noting that a total of 83 DEGs have involved in the plant hormone signal transduction pathway, which comprised the maximum number of the top 20 most significantly enriched KEGG pathways (Fig. 9). Moreover, a total of 32 DEGs were identified in auxin pathway, and most of them such as AUX/IAA and SAUR family members were significantly down-regulated, suggesting that *OsFtsH2* might participate in the regulation of auxin signaling pathway (Table S3). In addition, we also found that most genes associated with cytokinine, gibberellin and brassinosteroid signals were suppressed, especially the transcription factors *PIF4* and *BZR1*. These findings may give an explanation to the retarded growth in *osftsh2* mutants. However, the expression levels of most DEGs in the ethylene signaling pathway were up-regulated, which was consistent with the senescence phenotype in mutants. Anyhow the altered expression patterns of plant hormone signals could provide an insight into the molecular mechanism of *OsFtsH2* involved in the growth and development of rice seedlings.

### qRT-PCR validation of DEGs identified by RNA-seq

**Fig. 10:**
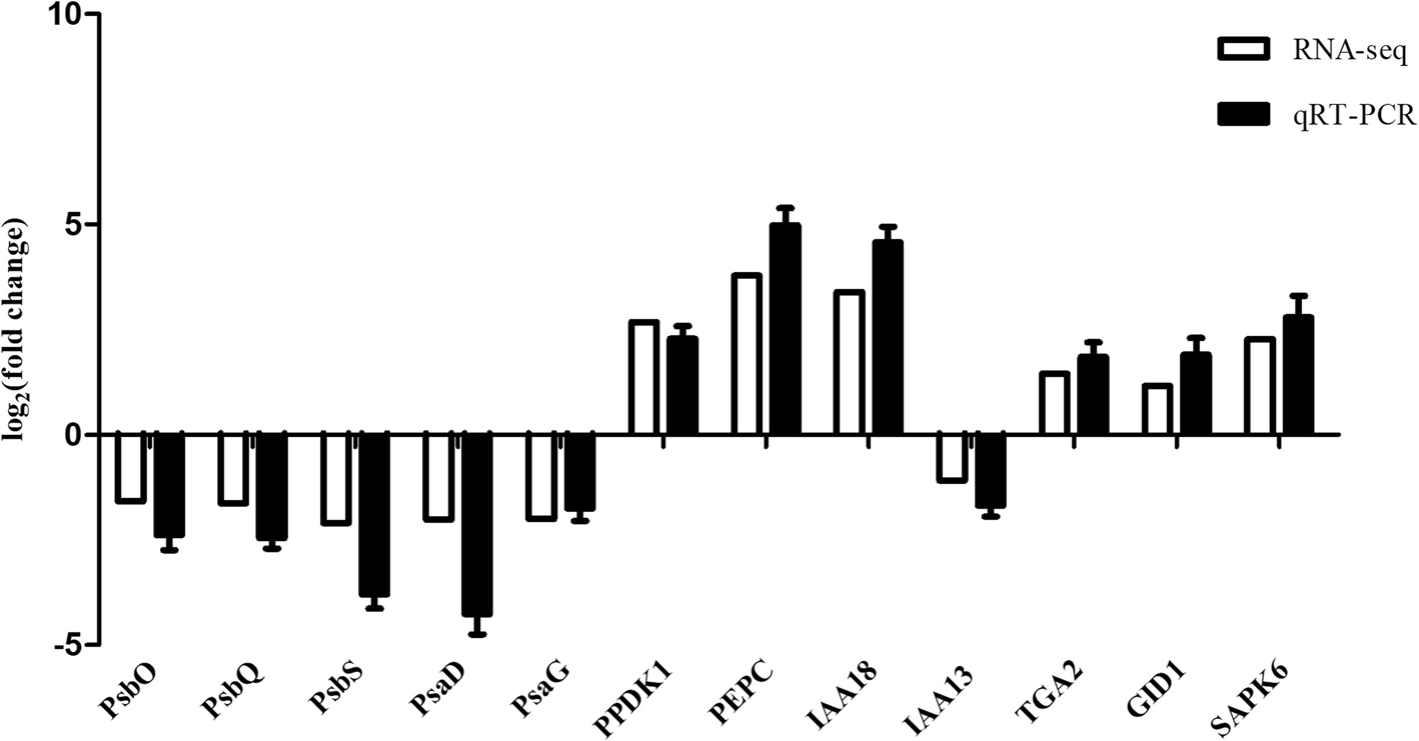
Validation of RNA-seq by qRT-PCR

To validate the RNA-seq transcriptome data, 12 genes associated with photosynthesis and plant hormone signals were selected for qRT-PCR analysis. The results showed that the expression tendency of these genes was basically consistent with the RNA-seq data (Fig. 10), verifying the reliability of these data.

## Discussion

### OsFtH2 plays a vital role in early development of chloroplast

Rice (*Oryza sativa*) contains nine FtsH genes in its genome [28, 42], and the function mechanisms of FtsH genes in rice have not been fully understood so far. In this study, we have successfully edited the coding region of *OsFtsH2* by CRISPR/Cas9 system to generate *osftsh2* knockout mutants (Fig. 3). These osftsh2 mutants exhibited phenotype of albino leaves and their contents of photosynthetic pigment were significantly less than that of wild type. The evaluation of photosynthetic parameters showed that *osftsh2* mutants lacked autotrophic ability for photosynthesis (Fig. 5), which was consistent with the lethal phenotype of their seedling. Subcellular localization demonstrated that the OsFtsH2 protein was targeted to chloroplast (Fig. 2), and no normal chloroplasts were formed in *osftsh2* mutants (Fig. 6). In addition, transcriptome analysis showed various genes related to photosynthesis were suppressed in *osftsh2* mutants (Table S2). Also OsFtH2 had the close evolutionary relationships with type B (AtFtsH2/8) chloroplastic FtsHs in *Arabidopsis thaliana* (Fig. 1A) and lacking of *AtFtsH2* caused a severe leaf variegation phenotype and *atftsh8* mutants have no visible phenotypes [21, 30–32]. Moreover, double mutants of *AtFtsh2AtFtsh8* showed an albino-like phenotype, suggesting that they had redundant functions to some extent and each one was required for chloroplast biogenesis [28, 33]. Considering the seedling lethal trait of *osftsh2* mutants, it can be concluded that OsFtsH2 performs a distinguishable function in chloroplast development and has no redundancy with other chloroplast localized FtsH genes in rice. All the results reveal that OsFtH2 plays a vital role in early development of chloroplasts in plants.

### OsFtH2 is crucial to biosynthesis in photosystem

Photosystem is one of the sites for light reactions to take place, which converts solar energy to chemical energy through energy transfer, photoelectric conversion and electron transfer. Previous studies demonstrated that chloroplast FtsH metalloproteases mainly participated in degrading the proteins at the photodamaged D1 reaction center during the PSII repair cycle, as well as facilitated to remove unassembled PSII subunits, light-harvesting complex II (LHCII) proteins and cytochrome b6 f Rieske FeS proteins proteins [17, 29, 36]. Interestingly, both *var1* (*atftsh2*) and *var2* (*atftsh5*) mutants had lower amount of PSI complex proteins under normal conditions of growth, and also had similar levels of PSII core protein D1 as compared with the wild type. In addition, either *var1* or *var2* mutants showed no differences in psaA/B transcript accumulation and translation as compared with the wild type, suggesting that later stages of PsaA/B protein expression were impaired in these mutants [48]. Similarly, *slr0228* encoded a chloroplast FtsH gene in *Synechocystis sp. PCC 6803*, and its disruption could triger a major reduction in the abundance of PSI without affecting the cellular content of PSII or phycobilisomes [16]. Moreover, the abundance of PsaA was significantly reduced in the *C. reinhardtii ftsh1–1* mutant under 50 or 150 mmol photons m^− 2^ s^− 1^ [49]. In one word, FtsH gene was required for the biosynthesis of PSI, which was evolutionarily conserved in oxygenic photosynthetic organisms [48]. In this study, we found that almost all genes of PSI and PSII subunits were greatly down-regulated in o*sftsh2* mutants (Table S2), and also the actual photochemical efficiency (ФPSII and ФPSI) was much lower in *osftsh2* mutants (Fig. S1). Thus, we can conclude that OsFtsH2 is crucial to biosynthesis of photosystem in rice. However, it is not clear whether OsFtsH2 is directly or indirectly involved in the assembly of the photosystem and what its substrates are. The specific mechanism needs further study in the future.

### OsFtH2 may influence chloroplast-to-nucleus signaling

Retrograde signaling established an important regulatory mechanism for chloroplasts to regulate their metabolism and development state through chloroplast-to-nucleus signaling [50]. ROS in chloroplast, could act as signaling molecules to regulate the transduction of chloroplast-to-nucleus signal [51]. Retrograde signaling was triggered in the *var2* (*atftsh2*) mutant through accumulation of singlet oxygen, which activated the unfolded/misfolded protein response to balance the FtsH2 deficiency [52]. Similarly, the contents of H_2_O_2_ and O_2_^−^ in *osftsh2* mutants were significantly increased as compared to that in wild type (Fig. 7). In addition, some chloroplast-localized nuclear genes such as RbcS genes and Lhcb genes were substantially suppressed in *osftsh2* mutants (Table S2). These results suggested that OsFtH2 would influence chloroplast-to-nucleus signaling in rice.

## Conclusions

In conclusion, three transgene-free homozygous and *OsFtsH2* knockout mutants were generated by CRISPR/Cas9 genome editing system. Phenotypic analysis revealed that these *osftsh2* mutants were albino seedlings and eventually died at three leaves stage. OsFtsH2 targeted to chloroplast and remained much higher levels of expression in green tissues. The observation by transmission electron microscopy showed that the ultrastructure of chloroplasts was severely impaired in *osftsh2* mutants, and the measurement of photosynthetic parameters verified that the net rate of photosynthesis in the mutants had negative values. Moreover, RNA sequencing analysis indicated that the expression of genes related photosynthetic pathways were seriously inhibited in *osftsh2* mutants. Overall, OsFtsH2 would play a vital role in early development of chloroplasts in rice.

## Materials and methods

### Plant materials and growth conditions

A japonica rice named as ‘Donjin’ was used for genetic transformation and physiological experiments in this study. The seeds of Donjin were provided by Zhejiang University, China. The germinated seeds of rice were grown in hydroponic solution according to the recommendation of the International Rice Research Institute. Rice seedlings were grown in growth chambers under a 12-h-light (30 °C)/12-h-dark (22 °C) photoperiod and the photon flux density of about 500 μmol m^− 2^ s^− 1^ as previously described [53].

### Plasmid construction and plant transformation

The CRISPR/Cas9 system was adopted to generate *osftsh2* mutants according to the previous procedure [54]. In brief, two sites of CRISPR/Cas9 target were selected from the first coding exon of OsFtsH2 in the CRISPR-PLANT database (www.genome.arizona.edu/crispr/), and then two cassettes of sgRNA expression were inserted into the CRSPR/Cas9 binary vector (pYLCRISPR/Cas9-MH) by Golden Gate cloning. Callus were derived from mature seeds of wild type rice and then transformed with the constructed CRISPR/Cas9 vector using the *Agrobacterium tumiefaciens* strain *EHA105* in accordance with a conventional method [55]. All of the primers are listed in Table S4.

### Analysis of protein sequence

Homologous protein sequences of OsFtsH2 were obtained by the BLASTP program (www.ncbi.nlm.nih.gov) and they were aligned using the DNAMAN software. The neighbor-joining phylogenetic tree was generated using the software MEGA 5.1. Bootstrap analysis with1000 replicates was applied to assess the significances of the nodes. Conserved motif analysis of FtsH2 proteins was performed by the MEME (http://meme-suite.org/) and SMART (http://smart.embl-heidelberg.de/) online program.

### Measurement of photosynthetic pigment and photosynthetic parameters

Photosynthetic pigment contents of leaves were determined according to the previously described method [56]. Fresh second top leaves (0.2 g) collected at 7-day-old seedling stage were cut into small pieces and then immersed in 5 mL of 95% ethanol for 48 h at room temperature under dark conditions. After residual debris was discarded by centrifugation, the supernatants were analysed with a spectrophotometric scanning (DU800, Beckman, Fullerton, USA) to detect absorption values at 663, 645 and 470 nm. Three biological replicates were analyzed for each sample.

Photosynthetic parameters of leaves in wild type plants and *osftsh2* mutants, including net photosynthetic rate (Pn), transpiration rate (Tr), stomatal conductance (Gs) and intercellular CO_2_ concentration (Ci), were measured using a portable photosynthesis system (Licor-6400, LI-COR, Lincoln, NE, USA) according to the manufacturer’s instructions. In the sample chamber, all measurements were performed at a photon flux density of 1000 μmol m^− 2^ s^− 1^, a leaf temperature of 30 °C and CO_2_ concentration of 400 μmol mol^− 1^. Each measurement was repeated three times and its value was averaged.

### Transmission electron microscopy

Analysis of transmission electron microscopy was conducted according to our previous procedure [57]. Briefly, a sample of the leaves was fixed in a solution of 2.5% glutaraldehyde and then in 1% osmium tetroxide at 4 °C. After fixation, the tissues were further dehydrated in gradient ethanol series and finally embedded in resin. Ultrathin sections (50 nm) were performed using a Leica EM UC7 ultra-microtome, and stained with uranyl acetate. Samples were observed under a Hitachi H-7650 transmission electron microscope.

### Subcellular localization of OsFtsH2

The full-length CDS sequence of OsFtsH2 without the termination codon was amplified, and then inserted into the modified pCambia1300-GFP vector with the CaMV35S promoter. The as-obtained OsFtsH2-GFP fusion vector was transformed into rice protoplasts as described previously [58], and the GFP fluorescence was determined by a florescence microscope (Zeiss LSM710). The OsFtsH2-GFP vector construction primers are listed in Table S4.

### Determination of H_2_O_2_ and O_2_^−^ production

Leaf samples of wild type plants and *osftsh2* mutants at 7-day-old seedling stage were collected for ROS content measurement. H_2_O_2_ and O_2_^−^ production in leaves were measured using reagent kits (Beijing Solarbio Science and Technology, China) according to the manufacturer’s instructions.

### RNA sequencing (RNA-seq) analysis

The total RNA of wild type plants and *osftsh2–1* mutants was extracted from their second leaves of 7-day-old seedlings. Each sample needed more than 1 μg of RNA to construct the RNA-seq libraries. The mRNA for sequencing was purified using poly(T) oligonucleotide-attached magnetic beads (Illumina, Inc., San Diego, CA, USA) and then was fragmented to small pieces about 300 bp. Random hexamer primers were used to synthesize the first-strand cDNA, and the second-strand cDNA was synthesized over DNA polymerase I and RNase H. Six RNA libraries were constructed and sequenced on an Illumina Novaseq 6000 sequencing platform (Majorbio, Shanghai). The sequencing data were analyzed according to a previous study [59]. RPKM (reads per kb per million reads) was used to describe the expression levels of genes, and genes with RPKM > 1 were considered to be expressed. The differentially expressed genes (DEGs) were assigned as |Log2(fold change) | > 1.0 and *p* values < 0.05. GO (Gene Ontology) enrichment analysis of DEGs was conducted with using agriGO web-based tools (http://bioinfo.cau.edu.cn/agriGO/analysis.php). KEGG (Kyoto Encyclopedia of Genes and Genomes) pathway enrichment analysis of DEGs was performed by the online KEGG web server (https://www.kegg.jp/).

### RNA extraction and qRT-PCR

Total RNA was extracted using an RNA Prep Pure Plant kit (TIANGEN, Beijing, China) according to the manufacturer’s instructions. First-strand cDNA was synthesized from 1 μg total RNA using Trans® Script One-Step gDNA Removal and cDNA Synthesis SuperMix (TransGen Biotech, Beijing, China). The qRT-PCR was performed with TransStart® Top Green qPCR SuperMix (TransGen Biotech, Beijing, China) using a LightCycler480 instrument (Roche, Sweden). The qRT-PCR reaction was carried out by incubation at 94 °C for 30 s followed by 40 cycles for 5 s and lasted at 60 °C for 30 s. The rice housekeeping gene (LOC_Os03g50885) was chosen as normalization control, and comparative expression levels were calculated by the 2^-ΔΔCT^ method. Three technical replicates on each of three biological replicates were conducted. All qRT-PCR primers are listed in Table S4.

## Supplementary Information

**Additional file 1: Fig. S1.** Chlorophyll fluorescence analysis of *osftsh2* mutants.

**Additional file 2: Fig. S2.** GO annotation analysis of DEGs in *osftsh2* mutants.

**Additional file 3: Table S1.** All DEGs in *osftsh2* mutants were listed.

**Additional file 4: Table S2.** DEGs in photosynthesis-related pathways.

**Additional file 5: Table S3.** DEGs in plant hormone signal transduction.

**Additional file 6: Table S4.** Primers sequence in this study were listed.

## Data Availability

The RNA-seq data has been submitted to the NCBI Sequence Read Archive (BioProject ID PRJNA735323, https://submit.ncbi.nlm.nih.gov/subs/bioproject/SUB9800680/overview). The datasets used and/or analyzed during the current study are available from the corresponding author on reasonable request.

## Abbreviations

BP: Biological process
CC: Cellular component
Ci: Intercellular CO_2_ concentration
DEGs: Differentially expressed genes
FtsH: Filamentation temperature-sensitive H
GO: Gene ontology
Gs: Stomatal conductance
KEGG: Kyoto encyclopedia of genes and genomes
LHCII: Light-harvesting complex II
MF: Molecular function
NEP: Nuclear encoded plastid RNA polymerase
PEP: Plastid-encoded RNA polymerase
Pn: Photosynthetic rate
PSII: Photosystem II
PSI: Photosystem I
RPKM: Reads per kb per million reads
ROS: Reactive oxygen species
Tr: Transpiration rate
UTR: Untranslated regions
WT: Wild type
