# Supplementary figures and images for: The essential roles of OsFtsH2 in developing the chloroplast of rice
Source: BMC Plant Biol. 2021 Oct 1;21:445. doi: 10.1186/s12870-021-03222-z (PMC8485545; doi:10.1186/s12870-021-03222-z)

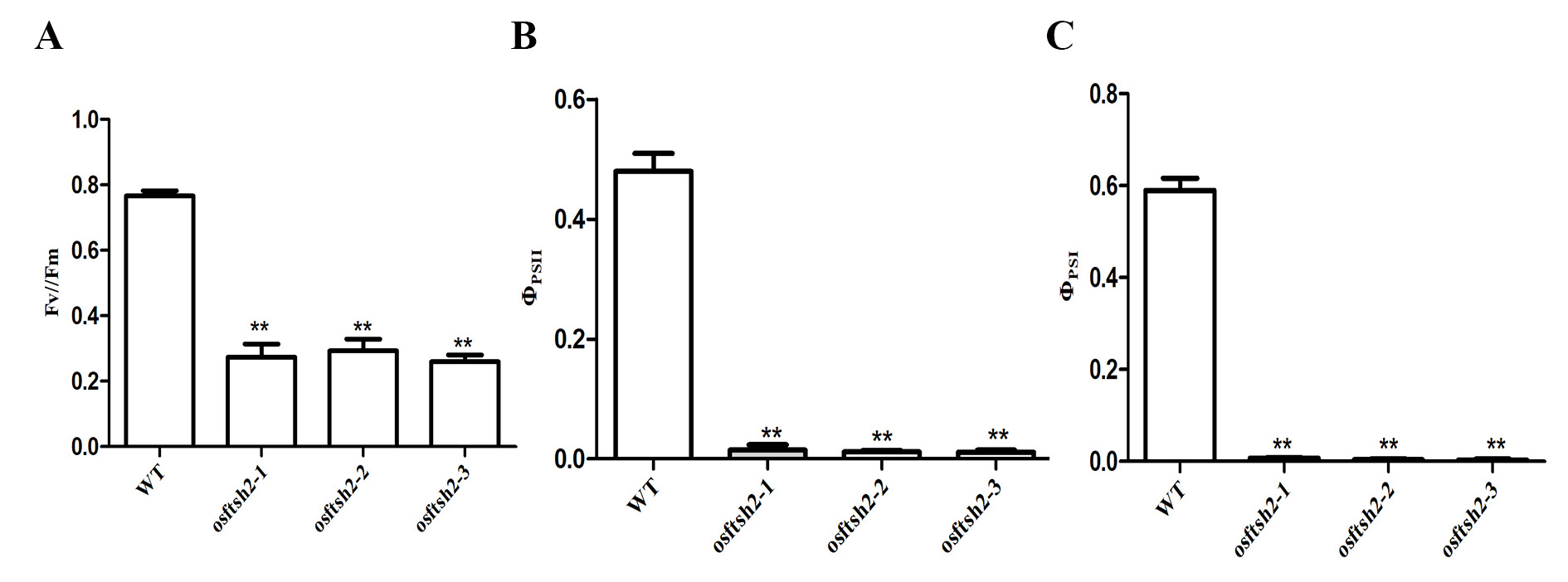

Supplement: Supplementary file 1 — Additional file 1: Fig. S1. Chlorophyll fluorescence analysis of osftsh2 mutants. [file 12870_2021_3222_MOESM1_ESM.jpg]

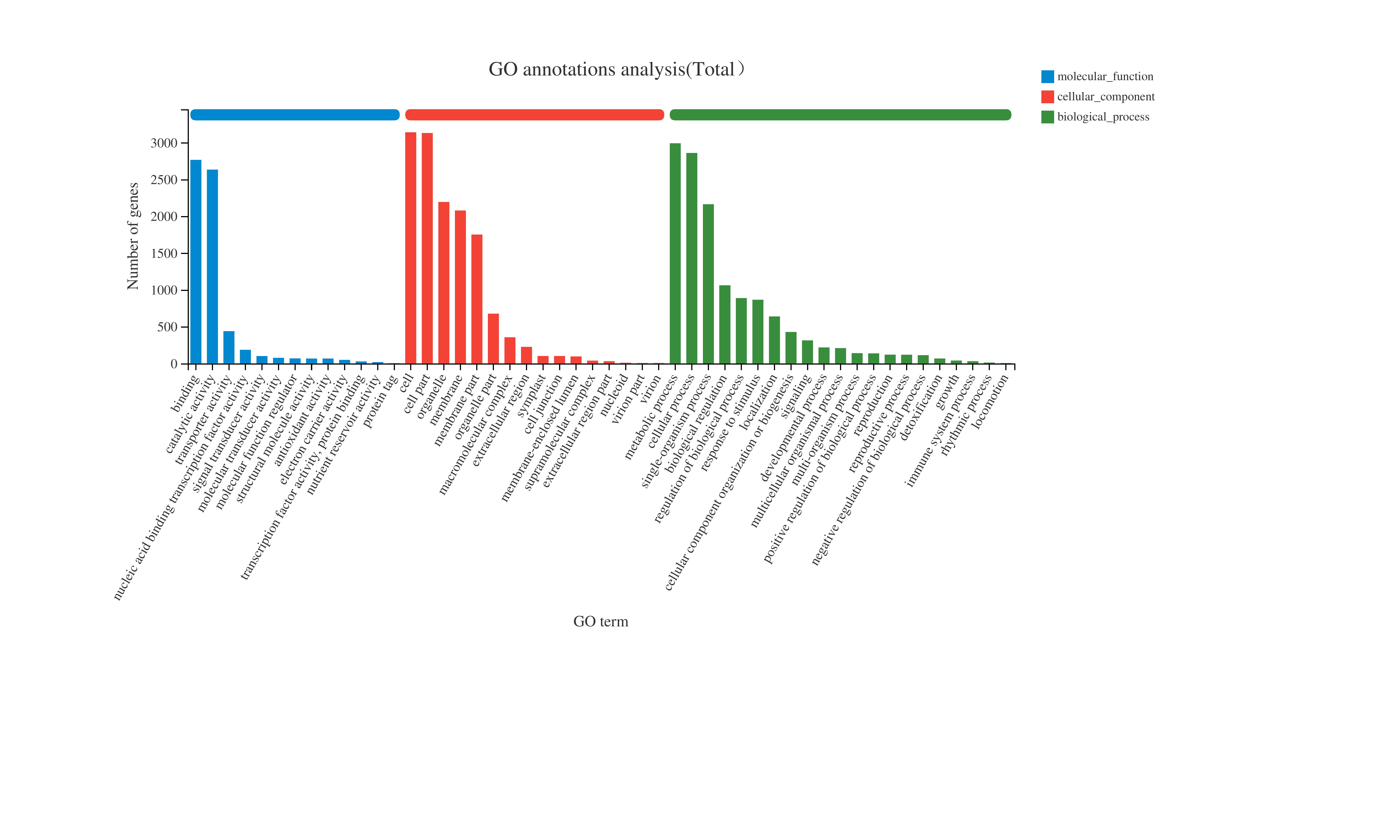

Supplement: Supplementary file 2 — Additional file 2: Fig. S2. GO annotation analysis of DEGs in osftsh2 mutants. [file 12870_2021_3222_MOESM2_ESM.jpg]
